# Vitality form expression in autism

**DOI:** 10.1038/s41598-020-73364-x

**Published:** 2020-10-14

**Authors:** L. Casartelli, A. Cesareo, E. Biffi, G. C. Campione, L. Villa, M. Molteni, C. Sinigaglia

**Affiliations:** 1 Scientific Institute IRCCS E.MEDEA, Child Psychopathology Department, Theoretical and Cognitive Neuroscience Unit, Bosisio Parini Lecco, Italy; 2 Scientific Institute IRCCS E.MEDEA, Bioengineering Lab, Bosisio Parini Lecco, Italy; 3 Scientific Institute IRCCS E.MEDEA, Child Psychopathology Department, Bosisio Parini Lecco, Italy; 4 Università Degli Studi Di Milano, Department of Philosophy, Via Festa del Perdono 7, 20122 Milano, Italy; 5 Cognition in Action (CIA) Unit, PHILAB, 20122 Milan, Italy

**Keywords:** Psychology, Human behaviour

## Abstract

The notion of “vitality form” has been coined by Daniel Stern to describe the basic features of action, which may reflect the mood or affective state of an agent. There is general consensus that vitality forms substantiate social interactions in children as well in adults. Previous studies have explored children with Autism Spectrum Disorder (ASD)’s ability in copying and recognizing the vitality forms of actions performed by others. In this paper we investigated, for the first time, how children with ASD express different vitality forms when acting themselves. We recorded the kinematics of ASD and typically developing (TD) children while performing three different types of action with two different vitality forms. There were two conditions. In the *what* condition we contrasted the three different types of action performed with a same vitality form, while in the *how* condition we contrasted the same type of action performed with two different vitality forms. The results showed a clear difference between ASD children and TD children in the *how*, but not in the *what*, condition. Indeed, while TD children distinguished the vitality forms to be expressed by mostly varying a specific spatiotemporal parameter (i.e. movement time), no significant variation in this parameter was found in ASD children. As they are not prone to express vitality forms as neurotypical individuals do, individuals with ASD’s interactions with neurotypical peers could therefore be difficult to achieve successfully, with cascading effects on their propensity to be tuned to their surrounding social world, or so we conjecture. If this conjecture would turn out to be correct, our findings could have promising implication for theoretical and clinical research in the context of ASD.

## Introduction

Our actions may take distinct forms according to our mood or affective state. For instance, our grip can be vigorous or delicate, our caress can be gentle or rushed. These action forms have been variously conceived by different researchers^1–3^. Daniel Stern coined the term of “vitality forms” claiming that their expression and recognition substantiate social interactions in children as well in adults, providing them with a primordial way of relating to each other^4,5^. Vitality forms could therefore offer an almost unique opportunity to explore core social skills, even when their development is not typical^6^.

Autism spectrum disorder (ASD) is a neurodevelopmental condition with heterogeneous clinical manifestations. These include difficulties in the social domain, verbal and nonverbal communication, and patterns of restricted and repetitive behaviours^7,8^. Previous studies found that children with ASD show selective difficulties in imitating and recognizing vitality forms expressed by others^9–12^. For instance, children with ASD were observed to have more difficulty than typically developing (TD) children in performing an imitation task which requires copying the vitality form of an observed action (e.g. its being gentle or forceful), although they did not differ from TD children when performing an imitation task which requires copying the goal of an observed action^9,10^. A similar dissociation between vitality form and action goal was found in a recognition task. ASD and TD children had to judge two observed actions as the same or different with respect to their goals or their vitality forms. The results showed that both ASD and TD children were able to identify the goals of the observed actions. However, unlike TD children, ASD children mostly failed to recognize which vitality form characterized these actions^11^.

Although these findings suggest that vitality form may contribute to a better understanding of social interaction difficulties in ASD, there are no studies investigating the way in which children with ASD express their own vitality forms when acting themselves, rather than observing someone else acting. The main aim of this paper is to fill this gap.

**Figure 1:**
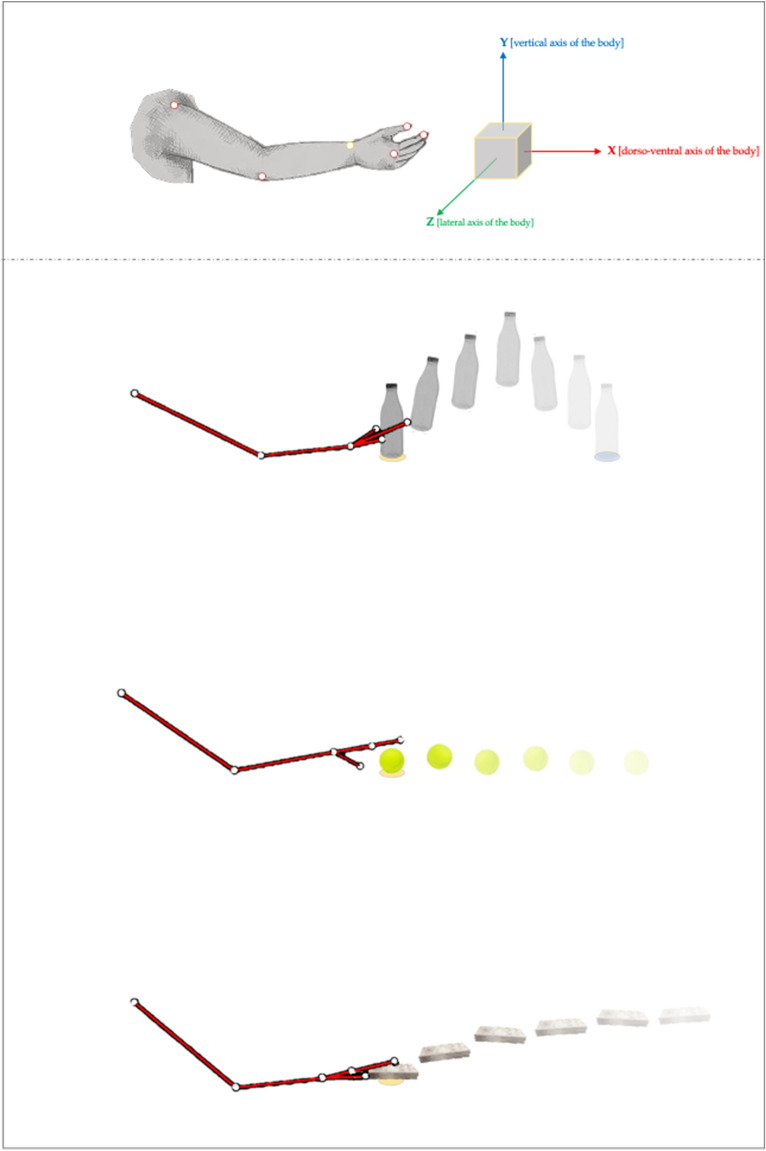
Upper panel. Marker-set and axes orientation. Schematic representation of the marker-set, with the wrist (radial styloid process, WRI) marker – the more stable and visible one used for the kinematic analyses—highlighted in yellow. In addition, graphical illustration of the axes orientation is reported. Bottom panel. Graphical illustration of the experimental set-up. Participants were requested to execute three *types of action* (from the top to bottom): (I) placing a bottle initially located in specific point (“object initial position”, yellow circle) on another point on the table (blue circle) along the forward axis (X axis, corresponding to the dorso-ventral direction of the body); (II) throwing a ball initially located in a specific point (“object initial position”, yellow circle) on the table along the forward axis (the ball has not to “bounce” on the table); (III) giving a packet of crackers initially located in a specific point (“object initial position”, yellow circle) along the forward axis (without placing it on the table). Note that the yellow circle was always the same for all types of action. At the beginning of each trial, participants had to place their right hand on the “hand starting position” (at a distance of 12 cm from the “object initial position”). All these three types of action were executed, alternatively, using two vitality forms: rude and gentle (in separate sessions in different days). Software used: Microsoft PowerPoint (Mac OS X), v.2020, www[point]microsoft[point]com Gimp (Mac OS X), v.2020, www[point]gimp[point]org.

According to Stern’s notion, vitality form primarily refers to *how* actions unfold in space and time, with each vitality form being characterized by a specific kinematic “contour”^5^. In order to assess whether and how such contours individuate action forms in autism, we compared upper limb kinematics of a group of 14 ASD children (3F; mean ± SD = 9.9 ± 1.6 years.months) to a control group of 14 TD children (5F; mean ± SD = 9.3 ± 0.8 years.months) (Table 1). Both groups were asked to perform three different manual actions, which were easy to compare from a kinematic point of view, that is, placing a bottle, throwing a ball, and giving a packet of crackers (without placing it) (see, Fig. 1). These actions had to be accomplished with two different vitality forms (i.e. *gentle* and *rude*) in two separate experimental sessions. Indeed, in a first session ASD and TD children had to perform the three different actions *gently*, while in a second session the same actions were performed *rudely*, or vice-versa.

**Table 1:** Descriptive statistics for ASD and TD group.

	ASD (N = 14; 3F)	TD (N = 14; 5F)	*p* Value
	Mean (SD)	Mean (SD)	
Age	9.9 (1.6)	9.3 (0.8)	.248
WISC III-*vocabulary*	8.8 (3.2)	10.9 (2.4)	.063
WISC III-*block design*	11.3 (2.9)	11.7 (3.2)	.713
Peabody	103.5 (17.2)	108.9 (12.4)	.347
ADOS–I / II / III / IV *module*	3/6/4/0	–	–
ADOS–*Social Interaction (S.I.)*	7.2 (2.2)	–	–
ADOS–*Language and Communication* (L&C)	4.8 (1.8)	–	–
ADOS–total (L&C + S.I)	12 (3.5)	–	–

Descriptive statistics comparing ASD and TD group for age and cognitive functioning, and ASD group’s characterization. Autism Diagnostic Observation Schedule (ADOS) is a structured observation that aims to test the presence of prototypical autistic behavioral phenotypes. It also provides specific cut-off to evaluate quantitatively the degree of such a presence. ADOS is structured to provide an interactive context between the patient and the clinician, in this way the clinician creates an environment in which autistic behaviors are more likely to happen. Although it is not required to reach the cut-off to make a diagnosis of ASD, it represents the widest shared instrument for supporting it, both in clinical and research settings. ADOS is a systematic and standardized tool to assess the presence of autistic behaviors; it is constituted by four (1st edition) or five (2nd edition) modules, each of them is designed with a specific protocol reflecting distinct ages and functional levels. The clinician, usually a child neuropsychiatrist or psychologist, has to evaluate the most appropriate module to be administered.

ASD and TD children’s kinematics was analysed in two different conditions: *what* and *how*, respectively. In the *what* condition we contrasted the three different types of action (placing, throwing *and* giving) performed with a same vitality form (gentle *or* rude), while in the *how* condition we contrasted the same action (placing, throwing *or* giving) performed with two different vitality forms (gentle *and* rude). To focus on potential differences in expressing vitality forms in ASD, one should expect to find any dissociation between groups in the *how* condition, and substantial similarities in the *what* condition. And this was what we actually found. The implication of this finding for better understanding social cognition in ASD will be discussed.

## Results

Upper limb kinematics of ASD and TD children were analysed by means of the following kinematic and spatiotemporal parameters: movement time (MT), peak velocity (pV), peak acceleration (pA), peak deceleration (pD), time to peak velocity (TpV), percentage of time spent in acceleration (T%Acc), percentage of time spent in deceleration (T%Dec), maximum displacements along the three Cartesian axes (MaxD_X_, MaxD_Y_, MaxD_Z_) (see also^13,14^). Concerning the Cartesian axes, X corresponds to the dorso-ventral axis of the body, Y to the vertical axis of the body, and Z to the lateral axis of the body (see Fig. 1). For each parameter, two indexes of *absolute differences* were computed: the first index measured the amount of variation of each parameter among the three different types of actions when performed with the same vitality form (hereafter, |*what*| index), whereas the second measured the amount of variation of each parameter when the same type of action was performed with the two different vitality forms (hereafter, |*how*| index). Intra-group and inter-group analyses were carried out.

### Intra-group analysis

#### Control group

The amount of variation of MT in the *how* condition (|*how*| index) was higher than in the *what* condition (|*what*| index), suggesting that this parameter was used by the control group mainly to modulate different vitality forms rather than different types of action (*p* = 0.005). The pV, pA and pD were also mainly used to modulate different vitality forms; indeed the |*how*| indexes were significantly higher than the |*what*| indexes for all three of these parameters (all *p* < 0.001). No differences between the two conditions emerged in the amount of variation of temporal parameters, such as TpV (*p* = 0.357), T%Acc (*p* = 0.816) and T%Dec (*p* = 0.545). This suggested that these parameters varied across different vitality forms to the same extent as they vary across different types of action. Amount of variation of MaxD_X_ was lower in the *how* (|*how*| index) than in the *what* condition (|*what*| index) (*p* = 0.007). Therefore, the amplitude of MaxD_X_ was mainly varied across different types of action performed with the same vitality form (*what* condition). No differences emerge between the two conditions for MaxD_Y_ (*p* = 0.592) and MaxD_Z_ (*p* = 0.567) (Table 2).

**Table 2:** Absolute indexes of modulation for the *how* and the *what* conditions.

Parameters	|*how*| index median (IQR)		|*what*| index median (IQR)		intra-group (*p *values |*how*|vs.|*what*|)		Inter-group (*p *values TD vs. ASD)	
	TD	ASD	TD	ASD	TD	ASD	|*how*|	|*what*|
MT (s)	0.276 (0.229)	0.195 (0.257)	0.187 (0.254)	0.194 (0.239)	**.005°**	.840	.124	.368
pV (mm/s)	1108.9 (851.5)	958.3 (792.0)	366.7 (537.1)	258.0 (456.8)	** < .001°**	** < .001°**	.202	.294
pA (mm/s^2^)	14,336.6 (11,632.2)	10,713.9 (8774.4)	5098.1 (8346.7)	3448.4 (7047.9)	** < .001°**	** < .001°**	.088	.374
pD (mm/s^2^)	24,024.2 (17,658.6)	15,065.8 (12,576.9)	8563.0 (9359.1)	4377.7 (10,446.1)	** < .001°**	** < .001°**	.014*	.963
TpV (s)	0.114 (0.145)	0.150 (0.202)	0.090 (0.172)	0.099 (0.155)	.357	.214	.458	.488
T% Acc (%)	20.133 (27.205)	11.672 (22.211)	18.572 (24.536)	20.026 (31.210)	.816	.080	.418	.444
T% Dec (%)	20.292 (29.511)	11.917 (22.275)	21.583 (24.225)	20.008 (31.242)	.545	.091	.359	.586
MaxD_X_ (m)	0.025 (0.044)	0.049 (0.066)	0.053 (0.056)	0.050 (0.051)	**.007°**	.901	.005*	.260
MaxD_Y_ (m)	0.069 (0.120)	0.061 (0.119)	0.077 (0.101)	0.064 (0.083)	.592	.899	.918	.213
MaxD_Z_ (m)	0.016 (0.024)	0.010 (0.021)	0.015 (0.022)	0.014 (0.018)	.567	.421	.229	.860

|*how*| index and |*what*| index for each parameter for both ASD and TD groups are reported. Values are expressed as median (IQR). *P *values of the intra-group analysis (|*how*| index Vs |*what*| index), and inter-group analysis (for the |*how*| index and for the |*what*| index, respectively) are also reported.

MT: movement time. pV: peak velocity. pA: peak acceleration. pD: peak deceleration. TpV: time to peak velocity. T%Acc: time spent in acceleration. T% Dec: time % spent in deceleration. MaxD_X_: Max Displacement along X axis.

MaxD_Y_: Max displacement along Y axis. MaxD_Z_: max displacement along Z axis.

|how| =|how| index; |what| =|what| index.

°*p *Values < .05, Mann Whitney U test |*how*| index vs. |*what*| index.

*p Values < .05, Mann Whitney U test TD vs. ASD.

Using “vitality form” as predictor, linear regression analysis showed that pV (R^2^ = 0.491, *p* < 0.001), pA (R^2^ = 0.465, *p* < 0.001), pD (R^2^ = 0.541, *p* < 0.001) and MT (R^2^ = 0.298, *p* < 0.001) were the only parameters explained with a moderate or reasonably high goodness of fit. On the contrary, when using “type of action” as predictor, no parameter was explained with R^2^ > 0.25. Extended results of linear regression analyses are reported in Supplementary Table S1.

#### ASD group

In the ASD group, the amounts of variation (|*how*| index Vs. |*what*| index) of most parameters were similar to the control group. More specifically, in the ASD group, the parameters pV, pA, and pD (all *p* < 0.001) vary between different vitality forms (*how* condition) more than they do between different types of action (*what* condition), as found in the TD group. Furthermore, as in the TD group, variations of temporal parameters such as TpV (*p* = 0.214), T%Acc (*p* = 0.080) and T%Dec (*p* = 0.091), were not significantly different between the two conditions. The same was found for MaxD_Y_ (*p* = 0.899) and MaxD_Z_ (*p* = 0.421), which did not vary differently between the two conditions. Strikingly, things were different for two critical parameters, that is, MT and MaxD_X_. Contrarily to the TD group, in the ASD group the amounts of variation of both MT (*p* = 0.840) and MaxD_X_ (*p* = 0.901) were not significantly different between the *how* and the *what* conditions (see, Table 2).

Using “vitality form” as predictor, linear regression analysis showed that pV (R^2^ = 0.493, *p* < 0.001), pA (R^2^ = 0.428, *p* < 0.001), pD (R^2^ = 0.445, *p* < 0.001) were the only parameters explained with a moderately high goodness of fit. Contrarily to what emerged for the TD group, “vitality form” does not explain the model of MT (*p* = 0.107). This means that “vitality form” explained pV, pA, and pD data in both groups in a comparable way, but MT was predicted only in the TD group. As in the TD group, when using “type of action” as predictor, no parameter was explained with an adequate goodness of fit (all R^2^ < 0.25). Extended results of the linear regression analyses are reported in Supplementary Table S1.

### Inter-group analysis

For each parameter, the |*how*| and |*what*| indices of the TD group were compared with those of the ASD group. This analysis enabled us to study differences between the two groups in the amount of variation of the spatiotemporal and kinematic parameters in each condition (*how* condition, *what* condition).

When considering the |*what*| index, no significant differences were found between the two groups (all *p* > 0.213). This indicates that there were no differences in the kinematic and spatiotemporal parameters between ASD group and TD controls that can be attributed to the execution of different types of action (*what* condition) (see Table 2). In contrast, some differences emerged between the two groups in the *how* condition. A higher variation of the pD parameter (*p* = 0.014) was reported for the TD group compared to the ASD group. The result of the pD parameter simply suggested a “quantitative” inter-group difference (i.e., the direction of modulation was the same for both groups, but it is higher in the TD one). This was also corroborated by regression analysis, in which “vitality form” is a regressor factor of pD parameter both for TD (R^2^ = 0.541; *p* < 0.001) and ASD (R^2^ = 0.445; *p* < 0.001) participants (Supplementary Table S1). Much more interestingly, a further difference between the two groups occurred for MaxD_X_, which was higher in the ASD group (*p* = 0.005). This was consistent with what we found in the intra-group analysis: in the TD group, a significant difference was reported between the *how* condition and *what* condition for this parameter, while in the ASD group no significant difference emerged (Table 2). No significant differences were reported between the two groups in the case of MaxD_Y_ (*p* = 0.918), MaxD_Z_ (*p* = 0.229) and temporal parameters such as TpV, T%Acc and T%Dec (all *p* > 0.359).

According to the intra-group analysis, computing the |*how*| index one could have expected an inter-group difference also in the modulation of MT, but this was not the case. Indeed, the intra-group analysis showed a significant difference between the *how* and *what* conditions in the TD group (*p* = 0.005), but this difference did not emerge in the ASD group (*p* = 0.840) (Table 2). However, exploring our dataset at the individual participant level, we noticed that the MT parameter was modulated both positively and negatively on varying the vitality form almost exclusively in ASD participants. Indeed, rude actions took more time than gentle actions to be executed in around 30% of cases in the ASD group (and in many other cases, gentle and rude actions took approximately the same time). By contrast, rude actions were basically shorter in time than gentle actions in TD participants, with MT_*rude*_ being longer than MT_*gentle*_ in less than 5% of cases. Because of this specific modulation in the ASD group, we decided to compute a further index (*how*_Sign_) taking into account the “direction” of the modulation in the *how* condition. Graphically, this modulation of the MT parameter was clearly shown by the individual distributions (“rain”) in the raincloud plots (Fig. 3, right panel). Consistently with the intra-group results, the *how*_Sign_ index showed a difference in the modulation of the MT parameter between TD and ASD participants (*p* = 0.002), with higher values in the TD group. As expected, results on MaxD_X_ parameters were consistent between the |*how*| index and the *how*_Sign_ index (Fig. 3, right panel; detailed results of the *how*_Sign_ index are reported in Supplementary Table S2). Finally, as anticipated above, linear regression analyses supported this result. Indeed, vitality form turned out to be a regressor factor of MT parameter for the TD group only (R^2^ = 0.298; *p* < 0.001) (Supplementary Table S1).

A warning should be reported. Caution is needed when dealing with data that should be replicated in further studies. This is generally true with any experimental approach, but it seems particularly urgent for kinematic and motion capture studies considering potential intra- and inter-subject(s) variability. Our results should therefore not be overestimated or oversimplified.

## Discussion

**Figure 2:**
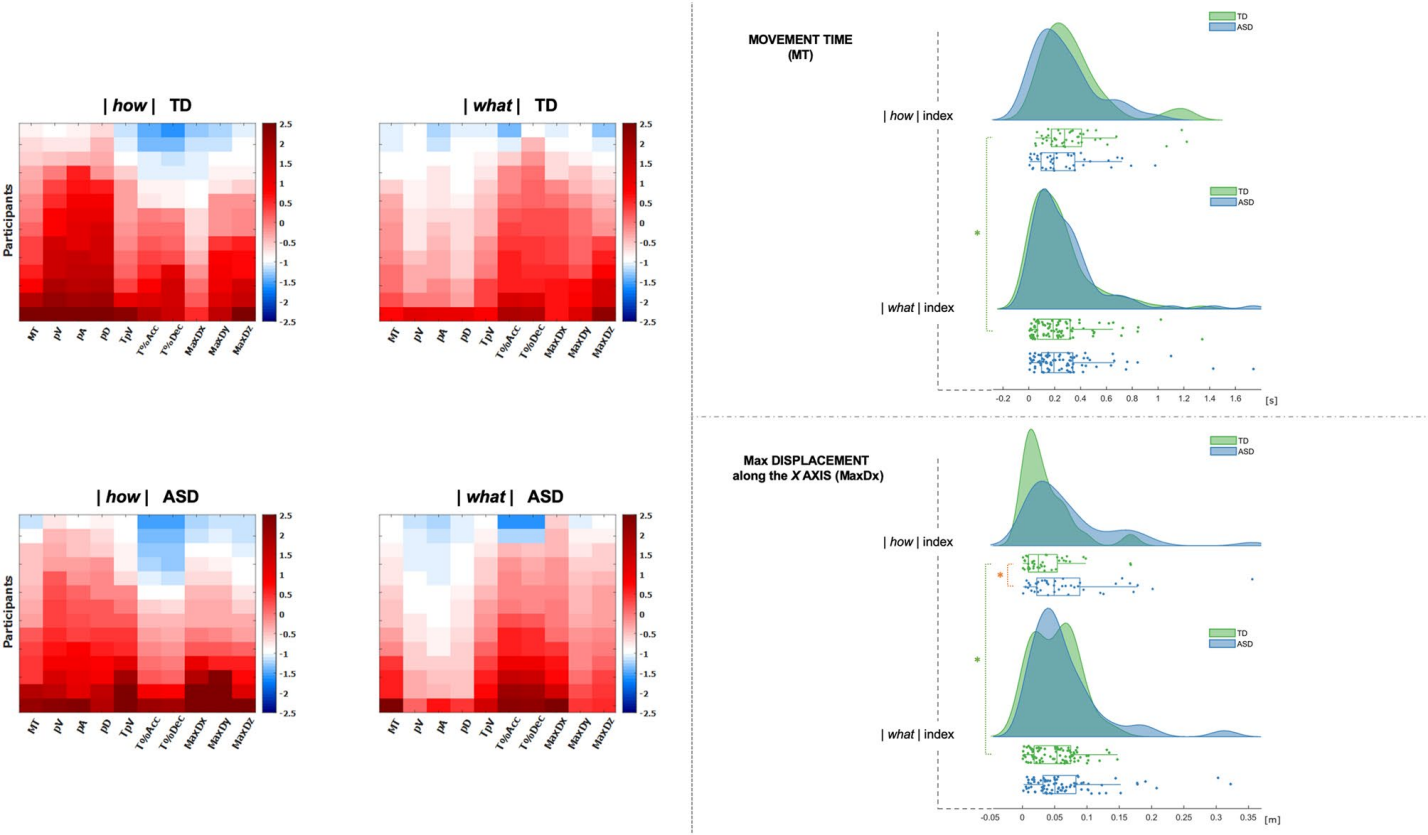
Left panel. Absolute indexes of modulation for the *how* and the *what* conditions. Heat maps showing amount of modulation (|*how*| index, left part; |*what*| index, right part) for participants (rows) and for each parameter (columns). Both TD group (top part) and ASD group (bottom part) are represented. Each cell represents the z-score of the absolute indexes computed through standardization, that allows to compare modulation of parameters based on different measurement units (e.g., spatial parameters, temporal parameters). Blue colors indicate low modulation (slight absolute differences), while red colors indicate high modulation (strong absolute differences) of the parameter. The z-scores are sorted within the columns in increasing order from top (slightest modulation) to bottom (strongest modulation); this facilitates the identification of patterns within each group but precludes the possibility to perform intra-subject comparison being the values relative to a single participant not on the same row (for details, see Supplementary Figure S1). Right panel. Absolute indexes of modulation for the *how* and the *what* conditions. Raincloud plots showing data distributions (“cloud”) and individual data jittered (“rain”) for Movement Time (MT) (top part) and maximum displacement along X axis (MaxD_X_; X axis corresponding to the dorso-ventral axis of the body) (bottom part) for the *how* condition (|*how*| index) and the *what* condition (|*what*| index), for both ASD (blue) and TD (green) groups. Box plots placed below the horizontal basis of the clouds represent the median value for each group (blue: ASD; green: TD), for both the *how* and the *what* conditions. Green asterisks represent statistically significant differences between conditions (*how* vs. *what*) within the TD group (intra-group analysis), and orange asterisk represents statistically significant difference between groups (inter-group analysis). Software used: Matlab, v.2018, www[point]mathworks[point]com.

The main aim of the present study was to investigate how children with ASD express their own vitality form when acting. ASD and TD children were asked to perform three different types of manual actions (placing, throwing and giving) with two different vitality forms (gentle and rude). Participants’ kinematics were generally found in both groups to be modulated more in the *how* (i.e. when varying vitality forms while holding the type of action constant) than in the *what* condition (i.e. when varying the types of action while holding vitality form constant). A graphical representation of this tendency is reported in Fig. 2 (left panel), in which heat maps are provided (for details, see *Statistical Analysis* Section). Regression analysis corroborates this finding, by showing that in both groups the explained variation in the data (i.e. R^2^ values) was globally higher when “vitality form” was selected as predictor (see, Supplementary Table S1). These and others similarities between TD and ASD participants’ kinematics, and mostly the absence of significant inter-group differences for any parameter in the *what* condition (Table 2), suggest not only that children with ASD understood our instructions trying to execute the three different types of action either gently or rudely, but also that our experimental setting was suitable for exploring possible differences in vitality form expression between ASD and TD children.

**Figure 3:**
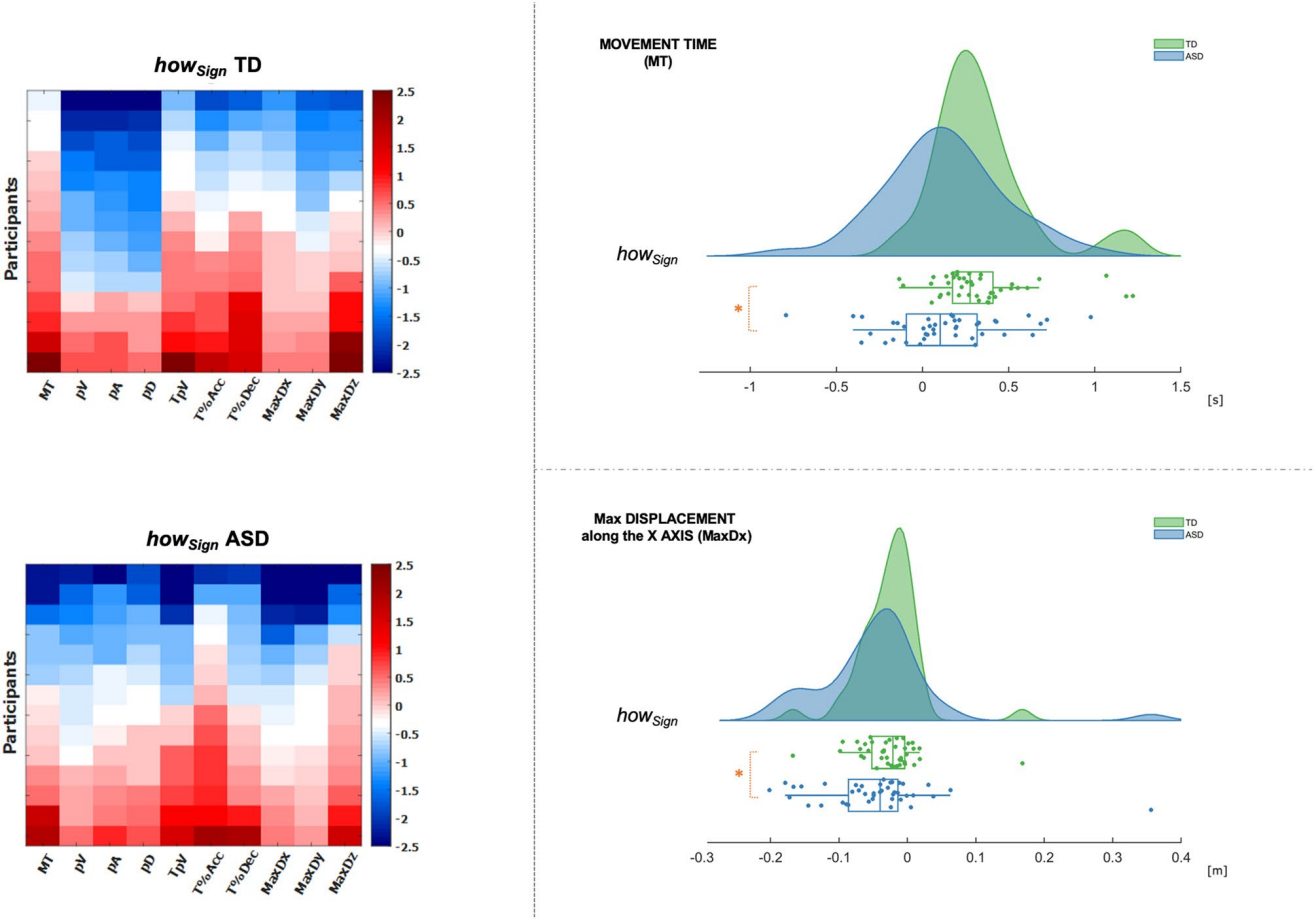
left panel. *How* condition taking into account the direction of the modulation. Heat maps showing the direction of modulation (*how*_Sign_ index) for participants (rows) and for each parameter (columns) of the *how* condition. Both TD group (top part) and ASD group (bottom part) are represented. Each cell represents the z-score of the *Sign* indexes computed through standardization, that allows to compare modulation of parameters based on different measurement units (e.g., spatial parameters, temporal parameters). Blue colors indicate negative modulation (Gentle < Rude) while red colors indicate positive modulation (Gentle > Rude) of each parameter. The z-scores are sorted within the columns in increasing order from top (negative modulation) to bottom (positive modulation); this facilitates the identification of patterns within each group but precludes the possibility to perform intra-subject comparison being the values relative to a single participant not on the same row (for details, see Supplementary Figure S1). Right panel. *How* condition taking into account the direction of the modulation. Raincloud plots showing data distributions (“cloud”) and individual data jittered (“rain”) for Movement Time (MT) (top part) and maximum displacement along X axis (MaxD_X_; X axis corresponding to the dorso-ventral axis of the body) (bottom part) for the *how* condition (*how*_Sign_ index), both for ASD (blue) and TD (green) groups. The *how*_Sign_ index takes into account the direction of modulation of each parameter in the *how* condition. Box plots placed below the horizontal basis of the clouds represent the median value for each group (blue: ASD; green: TD). Orange asterisks represent statistically significant difference between groups (inter-group analysis). Software used: Matlab, v.2018, www[point]mathworks[point]com.

The main finding was that, although a large number of parameters varied similarly in both groups, the spatiotemporal parameters MT and MaxD_X_ were *differently* modulated in ASD and TD children (with X axis indicating the extension from close to the body to away from the body; i.e. dorso-ventral axis of the body). Indeed, in TD children, MaxD_X_ significantly varied more in the *what* condition compared to the *how* condition, while MT significantly varied more in the *how* condition, reflecting the difference in expressing vitality forms. Strikingly, children with ASD did not present any significant variation in either parameters (MaxD_X_ and MT) between the conditions (Table 2). This was also reflected at the inter-group level. Indeed, while no inter-group difference was reported using the |*what*| index for any spatiotemporal and kinematic parameters (Table 2), significant differences emerged in the *how* condition (|*how*| index, Fig. 2, right panel; *how*_*Sign*_ index, Fig. 3, right panel. See also, Supplementary Table S2). In other words, both groups performed the different types of action in a very similar way, but results were different when we compared expression of vitality forms, suggesting a clear dissociation between TD and ASD participants that seems not to be related to generic differences in motor performance only.

It is true that there is a certain clinical agreement regarding a somehow generic reference to atypical aspects of motor functioning in ASD. This is certified by classical studies in the literature^15,16^ and by the introduction in the DSM-5 of motor functioning atypicalities as an *associated feature* of ASD (even if – notably – they are not *diagnostic criteria* for ASD, see^17^). It is also true that recent literature exploring motor functioning in infancy and childhood has not found a general consensus in ascertaining motor dysfunctions in ASD, but instead describes a complex picture of similarities and differences as compared to neurotypical controls^18–24^.

The presence of generic motor dysfunctions in ASD may bring our results into question. After all, it would be meaningless to compare vitality form expression between TD and ASD children if ASD kinematics was unreliable or a priori unstable. However, there are two reasons, at least, that make us confident in ruling out the view that general motor functioning difficulties in ASD could fully explain our results. First, if generic motor functioning difficulties in ASD were so pervasive as to impact severely even in these very easy daily-life types of action, one should expect random or pseudo-random inter-groups differences in many parameters in the *what* condition. But this was not the case (see Table 2; Fig. 2, right panel). Second, beyond the critical parameters MT and MaxD_X_ mentioned above, a number of parameters were modulated similarly in both groups also in the *how* condition, as also corroborated by regression analysis (Supplementary Table S1). The similar kinematic trends in these parameters not only further suggest that generic motor functioning difficulties in ASD cannot fully account for our results, but indirectly attest that children with ASD understood both the types of action to be executed and the vitality forms to be expressed.

Taken together, our findings indicate that TD children took advantage of two different parameters in the two different conditions. In the *what* condition, the different types of action to be performed were mostly reflected by MaxD_X_ modulation (this parameter measured maximum displacement along the dorso-ventral axis of the body). This was not surprising, giving the type of actions all the participants had to perform, that is, moving a bottle to a fixed point, throwing a ball, and giving a packet of crackers (Fig. 1). In the *how* condition, TD children expressed different vitality forms by modulating a parameter such MT, which Stern himself pointed out as a characterizing feature of each vitality form “contour”^5^ (see also^25^). In contrast with TD children, participants with ASD did not capitalize on a specific parameter in expressing their vitality forms. Indeed, they modulated both parameters MaxD_X_ and MT in a similar way in the *what* and *how* conditions. Although ASD participants clearly understood our instructions and tried to implement different vitality forms (in line with their performance in the Peabody test; see, Table 1), nevertheless they did not appear to be able to disambiguate the *what* and *how* conditions in these two critical parameters. Indeed, in the *how* condition (same type of action with different vitality forms), ASD children’s kinematic strategies were similar to those identified in the *what* condition (different types of action with same vitality form).

How can this difference between TD and ASD children in vitality form expression be explained? A likely hypothesis is that, in contrast with TD, motor parameters in ASD would not be settled in such a way as to flexibly represent *what* and *how* to unfold an action to be performed, thus reflecting the action goal and one’s own internal mood, respectively. In other words, ASD participants would not represent the *how* independently of the *what*, that is, they would not represent vitality form as an action component different from an action goal. This hypothesis seems to be in line with previous evidence suggesting that children with ASD show selective difficulties in imitating actions with different vitality forms.

In a first study Hobson and Lee^9^ explicitly contrasted the *what* and the *how* by asking a group of children and youths with ASD and TD controls to imitate, after a delay, some observed actions performed with different vitality forms. For instance, participants had to imitate the experimenter holding a pipe rack against the upper part of his left shoulder and running a wooden stick across it with his right hand, as if he were playing a violin. The experimenter strummed the stick over the pipe rack in either a rude or a gentle way. The results showed that while the TD controls imitated both the *what* and the *how* of the observed actions, participants with ASD were able to imitate the goal of the action only. Indeed, they had no difficulties in applying the stick to the pipe rack, but mostly failed in reproducing the corresponding vitality form. Similar results have been obtained by a subsequent study in which ASD and TD participants were asked to imitate actions performed with different vitality forms^10^. Groups did not differ in their ability to copy the action goals, even when accomplishing the goals required relatively complex movements. On the contrary, their imitative performance was significantly different when the vitality forms had been copied, with participants with ASD showing difficulties in imitating the vitality forms of the observed actions, especially when they were incidental to accomplishing their goals.

If our hypothesis turned out to be right, the difficulties of participants with ASD in vitality form imitation would be likely to be due *primarily* to their inability to represent the *how* independently from the *what* when they have to perform an action themselves. This could also shed new light on vitality form *recognition* difficulties in ASD. Rochat and colleagues^11^ asked ASD and TD children to judge whether two observed actions were similar/different relative to either their vitality forms (*how* task) or their goals (*what* task). The results indicated a dissociation between the two tasks, with children with ASD exhibiting difficulties in the *how*, but not in the *what*, task.

It is tempting to link these reported difficulties concerning individuals with ASD in vitality form recognition to their differences in representing and expressing vitality forms. Indeed, there is evidence that vitality form recognition may elicit, in the observer’s brain, processes and representations similar to those involved in vitality form expression^26–28^. In particular, it has been shown that acting, imaging acting and observing someone else acting with a given vitality form share the recruitment of a dorso-central portion of the insula^27^, which is known to receive information on the affective state of an agent^29,30^ and to modulate the parieto-frontal circuits directly involved in representing and executing actions^31^.

Accordingly, observing someone else acting may involve a transformation of the sensory information concerning the observed vitality form into processes and representations which would occur if the observer were expressing that vitality forms herself, and this would allow her to recognize it, by tracking another’s corresponding mood^32^. However, our data indicate that individuals with ASD do not represent and express their vitality forms as neurotypical individuals do. So, when observing someone else acting, they may be less inclined to rely on their own processes and representations of the corresponding vitality forms. This could explain why they had difficulties in recognizing them when expressed by others.

It is worth noting that motor cognition (action execution and recognition, and their mutual relationship) have been shown to be anomalous in ASD by previous studies (for a review,^33^). For instance, Cattaneo and colleagues^34^ found that children with ASD did not represent motorically a sequence of basic actions (e.g. reaching-grasping-putting into the mouth) as chained to each other in virtue of their means/end relations (reaching *for* grasping *for* putting into the mouth) both when they were performing those actions themselves and also when they were observing someone else performing them (see also^35,36^). Strikingly, Boria and colleagues^37^ showed that children with ASD had no difficulties in identifying single action goals such as taking a glass (see also^38^), but they did show difficulties in understanding *why* those actions had been performed (e.g. taking a glass *for* drinking or moving away). These difficulties were especially pronounced when understanding required taking into consideration specific motor cues (i.e. object affordances).

Overall, these results suggest children with ASD’s difficulties in action understanding may be tightly related to the different way through which they motorically represent their own actions. Our findings not only extend this picture to the vitality form dimension, but also pave the way to a better characterization of individuals with ASD’s peculiarities in social interaction. As already mentioned, vitality forms have been recognized as a key element of primary intersubjectivity, deeply shaping our experience of both ourselves and others^1–6^. As they are not prone to represent and express vitality forms as neurotypical individuals do, individuals with ASD’s interactions with neurotypical peers could therefore be difficult to achieve successfully, with cascading effects on their propensity to be tuned to their surrounding social world.

This does not fully account for the complex pathophysiology and heterogeneous phenotypical manifestations of ASD, of course. Nevertheless, our findings point to a key feature of primary social interaction which has been largely ignored in the ASD literature. Although additional research is needed, vitality form expression represents a promising building block for a deeper understanding of individuals with ASD’s peculiarities in social cognition.

## Methods

### Participants

Thirty children took part to the experiment but two children of the ASD group did not complete the experimental session due to loss of compliance. The final sample include 14 children for the ASD group (3F; mean ± SD = 9.9 ± 1.6 years.months), and 14 children (5F; mean ± SD = 9.3 ± 0.8 years.months) for TD group. Left-handed individuals, as indicated by a questionnaire adapted from the Edinburgh Handedness Inventory^39^ for Italian native speakers, were excluded.

Inclusion criteria for the ASD group were: (1) full scale IQ ≥ 70 as measured by the Italian version of Wechsler Intelligence Scale for Children-Revised (WISC-III,^40^); (2) absence of pharmacological therapy; (3) normal or corrected-to-normal vision and hearing; (4) absence of co-diagnosis of Developmental Coordination Disorder (CDC) or other major behavioural disorders (e.g., Conduct Disorder, etc.); (5) absence of any other neurological and psychiatric comorbidity. ASD participants were recruited at the Child Psychopathology Unit of the Scientific Institute IRCCS MEDEA (Bosisio Parini, Lecco, Italy). Clinical diagnosis of ASD was made by licensed clinicians with specific experience in the assessment of ASD in terms of DSM-IV diagnostic criteria and the Autism Diagnostic Observation Scale (ADOS). For the control group, parents reported no prior history of psychiatric or neurological disorder. TD participants’ cognitive level was screened with 2 subtests of the WISC-III that test verbal and visuo-perceptual abilities (i.e., vocabulary, block design). No statistically significant difference between groups is reported neither for block design (t_(26)_ = 0.37, *p* = 0.71) nor for vocabulary (t_(26)_ = 1.94, *p* = 0.06) subtest. Comprehension receptive skills were also tested with the Peabody Picture Vocabulary Test (PPVT-R), and performance do not differ between ASD group and controls (t_(26)_ = 0.96, *p* = 0.35) (see Table 1).

The protocol was approved by the local ethical committee (Scientific Institute IRCCS E. Medea) and was conducted according to the Declaration of Helsinki. Participants’ parents or legal guardian(s) signed the informed consent.

### Task and procedure

The experiment was conducted in a quiet room, where participants were seated at a table facing the experimenter (always the same, LC) with their right hand resting on a fixed point of the table. They were asked to perform three different types of action with their right hand (see also, Fig. 1):

- (I) placing a bottle initially located in a specific point (“object initial position”) on another point on the table along the forward axis (X axis, corresponding to the dorso-ventral direction of the body);
- (II) throwing a ball initially located in a specific point (“object initial position”) on the table along the forward axis (the ball has not to “bounce” on the table);
- (III) giving a packet of crackers initially located in a specific point (“object initial position”) along the forward axis (without placing it on the table).

Each action was split into two motor acts:

- (a) the first one, from the “hand starting position” to the “object initial position”, correspondent to the reaching phase;
- (b) the second one, from the “object initial position” to its end position, correspondent to the final phase of the action;

Actions belonged to the participants’ motor repertoire. Moreover, they have been selected among a group of daily life ones from previous studies (for similar protocols, see^26,27^), and accordingly to a pilot study, which confirmed that those ones were the most suitable in our experimental setting (i.e., more reliable data recording, see also “Data Analysis” section). All objects were initially located (= “object initial position”) at a distance of 12 cm from the starting position (= “hand starting position”). All these three types of action were executed, alternatively, using two vitality forms: rude and gentle (in Italian: “energico” and “delicato”, respectively).

Each action was performed six times for each vitality form, for a total of thirty-six trials (3 types of action × 2 vitality forms × 6 repetitions). To improve children’s compliance and data reliability, the experimental procedure was split into two sessions (18 trials for session), one for each vitality form, carried out on different days. Critically, from the one side this also minimized the risk of kinematic “dragging” effects (i.e., to make the *gentle* ruder or the *rude* more gentle); from the other it made very unlikely the possibility that one participant may express both rude and gentle vitality form in the same trial or she/he could be confused about the experimental request.

Order of presentation of both the types of action and vitality forms was randomized within and between participants. At the beginning of each session, all (both TD and ASD) participants were familiarized with the experimental procedure by the same experimenter (LC) that—in order to avoid any imitative effects—never performed any actions and never stated phrase such as “this is gentle” or “this is rude”. In addition, he never used any terms that may be a bias for our analysis (e.g. “fast”, “slow”, “rapid”, “strong”, etc.). Generally speaking, the experimenter avoided any *positive* description of the experimental requests (three types of action and two vitality forms, respectively). In the “types-of-action-familiarization” phase, he pushed participants to do actions by using verbal hints (e.g., “try again”). Noteworthy, in this familiarization phase it did not matter the vitality form, whereas we exclusively focused on the *type of action* executed by participants. In the “vitality-form-familiarization” phase, the experimenter familiarized with participants to verbally evoke their (kinematic) expression of rude/gentle vitality form. Distinct objects (e.g., a cup; a scotch roll) and distinct requests (e.g., to *drug* a cup; to *rotate* the scotch roll) were provided in order to avoid any bias during the test session. Noteworthy, during this vitality-form-familiarization phase it did not matter the type of action, whereas we exclusively focused on the *how* of the action expressed by participants.

During the test session, after each trial participants returned with their right hand at the “hand starting position”, and the experimenter placed the right object expected for the next trial on the “object initial position”. All participants clearly comprehended the experimental requests, as indirectly corroborated by kinematic similarities for many parameters between groups (all ones in the *what* condition, all but three parameters in the *how* condition) and by regression analyses, and performed the task generally well (e.g., 1–2 errors such as *placing the ball* instead of *throwing the ball*) or very well (no errors). Furthermore, 2D video recordings of the experimental sessions were also checked to verify compliance.

### Equipment and kinematics recording

Participants’ right upper limb kinematics was recorded using an optoelectronic system (OEP system-BTS Bioengineering, Milano, Italy) able to detect the 3D position and displacement of passive markers composed of adhesive plastic hemispheres, 10 mm in diameter, covered with reflective paper. This system consists of eight infra-red (IR) cameras, working at a sampling rate of 60 Hz, able to detect light reflected by the markers placed on specific body landmarks. Spatial resolution of the system is 0.3 mm. To maximize flexibility of analysis and minimizing potential data loss, we defined a marker-set of 6 markers placed, respectively, on the right arm acromion, elbow (lateral epicondyle), wrist (radial styloid process), third metacarpal head, fingernails of the index and of the thumb (Fig. 1, upper panel).

### Data analyses

Raw data were filtered by means of a triangular moving window filter (window duration of 66.7 ms)^13,14^. Being the main aim of the present study the investigation of potential differences between children with ASD and TD controls in expressing vitality forms, we analysed the kinematics of the second motor act in each type of action (placing a bottle, giving a packet of crackers, and throwing a ball). In fact, it is more reliable given that it excludes potential confounds related to the grasping phases of different objects. Preliminary analysis indicated that the most reliable marker for our aim was the one on the wrist (radial styloid process, hereafter WRI), as it was the more stable and visible in the second act recording. Thus, results reported in this paper focus only on the second act kinematics of the WRI-marker.

The onset of both acts was considered the first frame where the displacement of the WRI-marker along any axis (X,Y,Z) exceeds the value of spatial resolution of the system (0.3 mm). To determine the end of the reaching phase, we calculated the first frame following the onset in which the X, Y and Z displacements of the WRI-marker were all less than 0.3 mm (for similar approaches, see^41–43^). In our experimental setting, X axis corresponds to the dorso-ventral axis of the body, Y to the vertical axis of the body, and Z to the lateral axis of the body (Fig. 1).

The following parameters of the WRI-marker for the second act were computed: movement time (MT), peak velocity (pV), peak acceleration (pA), peak deceleration (pD), time to peak velocity (TpV). The acceleration phase was defined as the portion of movement from the movement onset up to pV. The deceleration phase is defined as the portion of movement from pV to the movement offset. Subsequently, we computed the percentage of acceleration time (T%Acc), defined as the duration of the acceleration with respect to MT, and the percentage of deceleration time (T%Dec) defined as the duration of the deceleration with respect to MT. Maximal displacements of the WRI-marker along the three Cartesian axis were also calculated. Maximal displacements of the WRI-marker, MaxD_X_, MaxD_Y_, MaxD_Z_, were defined as the maximum values of the rectified wrist marker track, within the time interval between the beginning and the end of the second act, along the *X*-axis, *Y*-axis, and *Z*-axis respectively.

For each participant and for each parameter, we computed the average values (mean ± standard deviation, SD) over the 6 trials performed for each type of action/vitality form: “ball-rude” (Ball _R_), “ball-gentle” (Ball _G_), “crackers-rude” (Crackers _R_), “crackers-gentle” (Crackers _G_), “bottle-rude” (Bottle _R_), “bottle-gentle” (Bottle _G_). The average values of each parameter were then used for further analysis. In particular, we considered the *what* condition (different types of action while holding vitality form constant) and the *how* condition (different vitality forms while holding the types of action constant). To analyse the amount of variation of each parameter in the two different conditions (*what* and *how*), we built distinct indexes. The index of absolute differences for the *what* condition (|*what*| index) measured the amount of variation of the parameter of interest among the different types of action, when performed with the same vitality form (rude or gentle). For each participant and each parameter, the absolute differences between different types of action performed with the same vitality form were computed. To recap, the following combinations were computed for each parameter (*p* = parameter, R = rude and G = gentle):

$$\left|{P\_Ball }_{ R}- {P\_Bottle}_{ R}\right|$$

$$\left|{P\_Ball }_{ R}-{P\_Crackers}_{ R}\right|$$

$$\left|\mathrm{P}\_{Bottle}_{ R}-{P\_Crackers}_{ R}\right|$$

$$\left|{P\_Ball }_{G}- {P\_Bottle}_{ G}\right|$$

$$\left|P{\_Ball}_{ G}- {P\_Crackers}_{ G}\right|$$

$$\left|{P\_Bottle }_{ G }- {P\_Crackers}_{ G}\right|.$$

Thus, the |*what*| index for each parameter was obtained computing the median value (Interquartile Range, IQR) over all the combinations and participants (14 for the TD and 14 for the ASD group, respectively).

The index of absolute differences for the *how* condition (|*how*| index) measured the amount of variation of the parameter of interest on varying the two vitality forms, when the same type of action was performed. For each participant and each parameter, the absolute differences between the rude (R) and gentle (G) vitality form for each of the three types of action were computed. To recap, the following combinations were computed for each parameter (P):

$$\left|{P\_Ball}_{ G}-{P\_Ball}_{ R}\right|$$

$$\left|{P\_Crackers}_{ G}-{P\_Crackers}_{ R}\right|$$

$$\left|{P\_Bottle}_{ G}-{P\_Bottle}_{ R}\right|.$$

Thus, the |*how*| index for each parameter was obtained computing the median value (IQR) over all the combinations and participants (14 for the TD and 14 for the ASD group, respectively). The |*how*| index measured the amount of variation of each parameter on varying the vitality forms without taking into account the “direction” of such variation (e.g., MT is greater in the gentle or in the rude actions). Thus, we also computed an index that included differences with sign (i.e., *how*_Sign_ index) to assess how each parameter was modulated on varying the vitality forms and to clarify the direction of the modulation.

### Statistical analysis

#### Intra–group analysis

To test differences between *how* condition and *what* condition, the Mann–Whitney U test was performed within each group, comparing the |*how*| and the |*what*| indexes for each parameter. This analysis led to identify, in each group, which were the spatiotemporal parameter(s) more relevant to modulate the kinematics of different vitality forms (*how* condition) or different types of action (*what* condition). Thus, when “|*how*| index >|*what*| index” for a specific parameter, it means that such a parameter encoded more the modulation of different vitality forms than the modulation of different types of action (see, Table 2; Fig. 2, right panel^44^). A significance level of 0.05 was used.

To evaluate the influence that vitality form and type of action have on each parameter, linear regression analyses were performed to model the relationship between variables, selecting “vitality form” or “type of action” as predictor. Such analysis allows to evaluate how these variables (i.e. “vitality form” and “type of action”) explain variation in spatiotemporal and kinematic data. Significant models (*p* < 0.05) with at least moderate goodness of fit (i.e., R^2^ > 0.29) were discussed (see, Supplementary Table S1).

#### Inter-groups analysis

To test inter-group differences between ASD and TD participants in the modulation of each spatiotemporal parameter to convey different vitality forms (*how* condition) and different types of action (*what* condition), the Mann–Whitney U test between groups was used. For each parameter, the |*how*| index and the |*what*| index were compared between the two groups in order to explore differences in terms of amount of variation (absolute difference) (Table 2; Fig. 2, right panel). Furthermore, to identify differences in the “direction” of variation for each parameter, *how*_Sign_ index of the TD group were compared with those of the ASD group. A significance level of 0.05 was used (see, Supplementary Table S2; Fig. 3, right panel).

#### Heatmaps

Heat maps showing amount of modulation (|*how*| index and |*what*| index, see Fig. 2, left panel) and direction of modulation (*how*_Sign_ index, see Fig. 3, left panel) per participant and per parameter were made with the Matlab heatmap function. Z-scores were obtained through standardization in order to compare modulation of parameters using different measurement units. To allow comparison between maps, the standardization for the absolute indexes was performed, for each parameter, merging all four datasets (|*how*|_TD, |*what*|_TD, |*how*|_ASD, |*what*|_ASD). Similarly, for the *Sign* indexes, the two datasets were merged to obtain z-scores (*how*_Sign__TD and *how*_Sign__ASD). Once the z-scores were obtained, values referring to conditions (*how* condition, *what* condition) and groups (ASD, TD) were plotted in different maps, with rows representing participants and columns representing parameters. We sorted the z-scores within the columns in increasing order from top to bottom; this facilitates the identification of patterns within each group but precludes the possibility to perform intra-subject comparison between different conditions, being the values relative to a single participant not on the same row (Supplementary Figure S1).

## Supplementary information

Supplementary file1

Supplementary file2

Supplementary file3

Supplementary file4

## Data Availability

Complete raw data, experiment codes, and more generally all materials and data supporting the findings of this study are available from the corresponding author upon reasonable request.

## References

[1] Trevarthen, C. The concept and foundations of infant intersubjectivity, in *Intersubjective Communication and Emotion in Early Ontogeny* 15–46 (Cambridge University Press, Cambridge, 1998).

[2] Trevarthen C, Aitken KJ (2001). Infant intersubjectivity: research, theory, and clinical applications. J. Child Psychol. Psychiatry Allied Discip..

[3] Rochat, P. *Others in Mind-Social Origins of Self-Consciousness*. (Cambridge University Press, Cambridge, 2009).

[4] Stern, D. N. *The Interpersonal World of the Infant*. (Basic Books, New York, 1985).

[5] Stern, D. N. *Forms of Vitality Exploring Dynamic Experience in Psychology, Arts, Psychotherapy, and Development*. (Oxford University Press, Oxford, 2010).

[6] Trevarthen, C. & Delafield-butt, J. T. Autism as a developmental disorder in intentional movement and affective engagement*. Front. Integr. Neurosci.* **7**, 49 (2013). 10.3389/fnint.2013.00049 PMC3713342 23882192

[7] Lai MC, Lombardo MV, Baron-Cohen S (2014). Autism. Lancet.

[8] Lord C, Elsabbagh M, Baird G, Veenstra-vanderweele J (2018). Autism spectrum disorder. Lancet.

[9] Hobson RP, Lee A (1999). Imitation and identification in autism. J. Child Psychol. Psychiatry Allied Discip..

[10] Hobson RP, Hobson JA (2008). Dissociable aspects of imitation: a study in autism. J. Exp. Child Psychol..

[11] Rochat MJ (2013). Impaired vitality form recognition in autism. Neuropsychologia.

[12] Di Cesare G (2017). Differences in action style recognition in children with autism spectrum disorders. Front. Psychol..

[13] Barbieri F, Buonocore A, Bernardis P, Dalla Volta R, Gentilucci M (2007). On the relations between affordance and representation of the agent’s effector. Exp. Brain Res..

[14] Campione CG, Piazza C, Villa L, Molteni M (2016). Three-dimensional kinematic analysis of prehension movements in young children with autism spectrum disorder: new insights on motor impairment. J. Autism Dev. Disord..

[15] Fournier KA, Hass CJ, Naik SK, Lodha N, Cauraugh JH (2010). Motor coordination in autism spectrum disorders: a synthesis and meta-analysis. J. Autism Dev. Disord.

[16] Gowen E, Hamilton A (2013). Motor abilities in autism: a review using a computational context. J. Autism Dev. Disord.

[17] ﻿American Psychiatric Association. *Diagnostic and Statistical Manual of Mental Disorders* (5th ed.). (2013).

[18] Mostofsky SH, Bunoski R, Morton SM, Goldberg MC, Bastian AJ (2004). Children with autism adapt normally during a catching task requiring the cerebellum. Neurocase.

[19] Landa R, Garrett-Mayer E (2006). Development in infants with autism spectrum disorders: a prospective study. J. Child Psychol. Psychiat..

[20] Iverson JM, Wozniak RH (2007). Variation in vocal-motor development in infant siblings of children with autism. J. Autism Dev. Disord.

[21] Focaroli V, Taffoni F, Parsons SM, Keller F, Iverson JM (2016). Performance of motor sequences in children at heightened vs. low risk for ASD: a longitudinal study from 18 to 36 months of age. Front. Psychol..

[22] Taffoni F, Focaroli V, Keller F, Iverson JM (2019). Motor performance in a shape sorter task: a longitudinal study from 14 to 36 months of age in children with an older sibling ASD. PLoS ONE.

[23] Achermann S, Nyström P, Bölte S, Falck-ytter T (2020). Motor atypicalities in infancy are associated with general developmental level at 2 years, but not autistic symptoms. Autism.

[24] Surgent OJ, Walczak M, Zarzycki O, Ausderau K, Travers BG (2020). IQ and sensory symptom severity best predict motor ability in children with and without autism spectrum disorder. J. Autism Dev. Disord..

[25] Di Cesare G, Stefani E, De Gentilucci M, De Marco D (2017). Vitality forms expressed by others modulate our own motor response: a kinematic study. Front. Hum. Neurosci..

[26] Di Cesare G (2014). The neural correlates of ‘vitality form’ recognition: an fMRI study. Soc. Cogn. Affect. Neurosci..

[27] Di Cesare G, Di Dio C, Marchi M, Rizzolatti G (2015). Expressing our internal states and understanding those of others. Proc. Natl. Acad. Sci..

[28] Di Cesare G (2016). Vitality forms processing in the insula during action observation: a multivoxel pattern analysis. Front. Hum. Neurosci..

[29] Craig AD (2002). How do you feel? Interoception: the sense of the physiological condition of the body. Nat. Rev. Neurosci..

[30] Craig AD (2014). Topographically organized projection to posterior insular cortex from the posterior portion of the ventral medial nucleus (VMpo) in the long-tailed macaque monkey. J. Comp. Neurol..

[31] DiCesare, G. *et al.* Insula connections with the parieto-frontal circuit for generating arm actions in humans and macaque monkeys. *Cereb. Cortex*, 1–8 (2018). 10.1093/cercor/bhy095 29741595

[32] Rizzolatti G, Sinigaglia C (2016). The mirror mechanism: a basic principle of brain function. Nat. Rev. Neurosci..

[33] Casartelli L, Molteni M, Ronconi L (2016). So close yet so far : Motor anomalies impacting on social functioning in autism spectrum disorder. Neurosci. Biobehav. Rev..

[34] Cattaneo L (2007). Impairment of actions chains in autism and its possible role in intention understanding. Proc. Natl. Acad. Sci. U. S. A..

[35] Fabbri-Destro M, Cattaneo L, Boria S, Rizzolatti G (2009). Planning actions in autism. Exp. Brain Res..

[36] Casartelli L, Federici A, Biffi E, Molteni M, Ronconi L (2018). Are we “motorically” wired to others? High-level motor computations and their role in autism. Neuroscientist.

[37] Boria S (2009). Intention understanding in autism. PLoS ONE.

[38] Hamilton AFC, Brindley RM, Frith U (2007). Imitation and action understanding in autistic spectrum disorders: how valid is the hypothesis of a deficit in the mirror neuron system?. Neuropsychologia.

[39] Oldfield RC (1971). The assessment and analysis of handedness: the Edinburgh inventory. Neuropsychologia.

[40] Wechsler D (1991). WISC-III: Wechsler Intelligence Scale for Children: Manual.

[41] Ferri F, Cristina G, Gentilucci M (2010). To me or to you When the self is advantaged. Exp. Brain Res..

[42] Ferri F, Campione GC, Dalla Volta R, Gianelli C, Gentilucci M (2011). Social requests and social affordances: how they affect the kinematics of motor sequences during interactions between conspecifics. PLoS ONE.

[43] De Stefani E (2014). The spatial alignment effect in near and far space: a kinematic study. Exp Brain Res.

[44] Allen, M., Poggiali, D., Whitaker, K., Marshall, T. R. & Kievit, R. A. Raincloud plots: a multi-platform tool for robust data visualization. *Wellcome Open Res*. **4**, 63 (2019). 10.12688/wellcomeopenres.15191.1 PMC6480976 31069261

