# Supplementary material for: Vitality form expression in autism
Source: Sci Rep. 2020 Oct 14;10:17182. doi: 10.1038/s41598-020-73364-x (PMC7560849; doi:10.1038/s41598-020-73364-x)
Supplement: Supplementary file 1 — Supplementary file1 [file 41598_2020_73364_MOESM1_ESM.pdf]

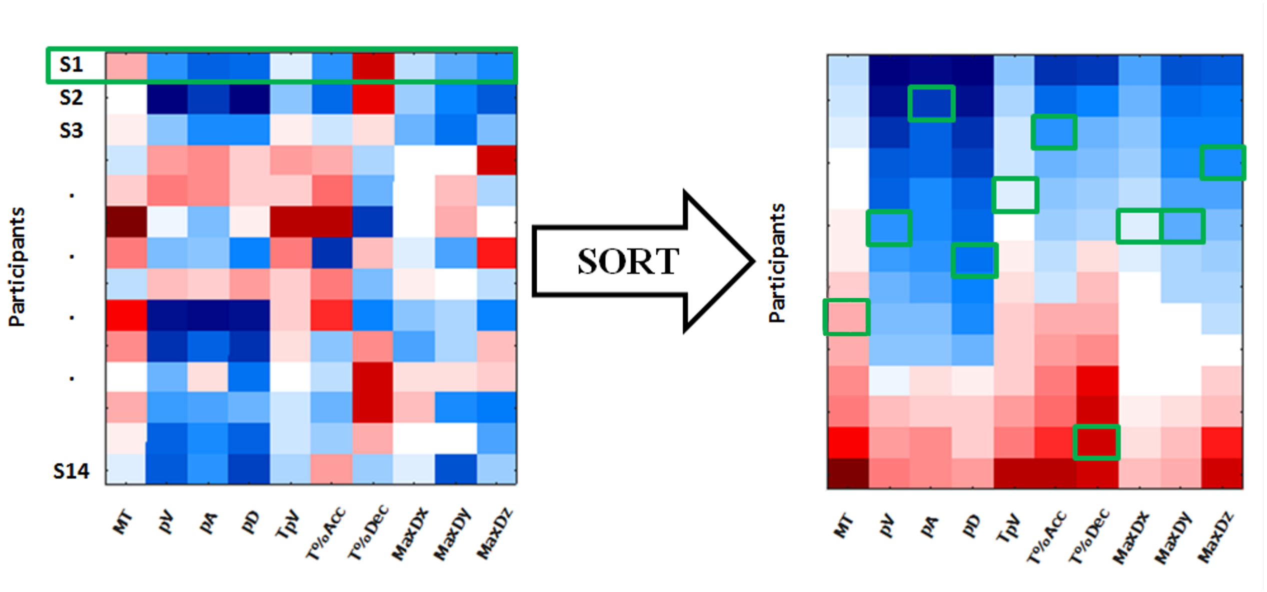

Supplementary Figure S1. Heat map sorting.

Z-scores into the heat maps were sorted within the columns in increasing order from top to bottom. This representation facilitates the identification of modulation patterns within each group but precludes the possibility to perform intra-subject comparison being the values relative to a single participant not on the same row.

Software used: Matlab, v.2018, www[point]mathworks[point]com Microsoft PowerPoint (Mac OS X), v.2020, www[point]microsoft[point]com
