# Supplementary material for: Vitality form expression in autism
Source: Sci Rep. 2020 Oct 14;10:17182. doi: 10.1038/s41598-020-73364-x (PMC7560849; doi:10.1038/s41598-020-73364-x)
Supplement: Supplementary file 2 — Supplementary file2 [file 41598_2020_73364_MOESM2_ESM.pdf]

SUPPLEMENTARY MATERIAL

TITLE PAGE

VITALITY FORM EXPRESSION IN AUTISM

L. Casartelli,[1*] A. Cesareo,[2*] E. Biffi,[2] G.C. Campione,[3] L. Villa,[3] M. Molteni,[3] C. Sinigaglia[4-5]

[1] Scientific Institute IRCCS E.MEDEA, Child Psychopathology Department, Theoretical and Cognitive Neuroscience Unit, Bosisio Parini (Lecco, Italy).

[2] Scientific Institute IRCCS E.MEDEA, Bioengineering Lab, Bosisio Parini (Lecco, Italy).

[3] Scientific Institute IRCCS E.MEDEA, Child Psychopathology Department, Bosisio Parini (Lecco, Italy).

[4] Department of Philosophy, Università degli Studi di Milano, 20122, Milan, Italy.

[5] Cognition in Action (CIA) Unit, PHILAB, 20122, Milan, Italy.

* co-first authorship
