# Supplementary material for: Vitality form expression in autism
Source: Sci Rep. 2020 Oct 14;10:17182. doi: 10.1038/s41598-020-73364-x (PMC7560849; doi:10.1038/s41598-020-73364-x)
Supplement: Supplementary file 3 — Supplementary file3 [file 41598_2020_73364_MOESM3_ESM.pdf]

Supplementary Table S1a. Results of linear regression analysis, using “vitality form” as predictor, for TD and ASD groups.

Variables	R^2	F	p-values
**TD Group**			
**MT [s]**	.298	34.885	<.001
**pV [mm/s]**	.491	79.251	<.001
**pA [mm/s^2]**	.465	71.241	<.001
**pD [mm/s^2]**	.541	96.512	<.001
$MaxD_X$ [m]	.093	8.443	.005
$MaxD_Y$ [m]	.164	16.137	<.001
$MaxD_Z$ [m]	.001	.041	.840
TpV [s]	.008	.655	.421
T% Acc [%]	.226	23.953	<.001
T% Dec [%]	.200	20.442	<.001
**ASD Group**			
MT [s]	.031	2.653	.107
**pV [mm/s]**	.493	79.733	<.001
**pA [mm/s^2]**	.428	61.407	<.001
**pD [mm/s^2]**	.445	65.745	<.001
$MaxD_X$ [m]	.104	9.535	.003
$MaxD_Y$ [m]	.150	14.490	<.001
$MaxD_Z$ [m]	.021	1.752	.189
TpV [s]	.037	3.121	.081
T% Acc [%]	.120	11.223	.001
T% Dec [%]	.119	11.113	.001

Results report proportion of variance in the dependent variables that can be explained by the independent variable (R^2), F-statistic (F) and the p-value associated with it.

MT: movement time. pV: peak velocity. pA: peak acceleration. pD: peak deceleration. TpV: time to peak velocity. T%Acc: time % spent in acceleration. T% Dec: time % spent in deceleration. $MaxD_X$: Max Displacement along X axis. $MaxD_Y$: Max Displacement along Y axis. $MaxD_Z$: Max Displacement along Z axis.

*Variables with R^2>=.29 and p-value<.05 are marked in **bold***

Supplementary Table S1b. Results of linear regression analysis using "type of action" as predictor, for TD and ASD groups.

Variables	R^2	F	p-values
**TD Group**			
MT [s]	.098	8.915	.004
pV [mm/s]	.033	2.767	.100
pA [mm/s^2]	.097	8.808	.004
pD [mm/s^2]	.023	1.901	.172
$MaxD_X$ [m]	.021	1.734	.192
$MaxD_Y$ [m]	.127	11.929	.001
$MaxD_Z$ [m]	.002	.142	.707
TpV [s]	.026	2.224	.140
T% Acc [%]	.230	24.541	<.001
T% Dec [%]	.239	25.776	<.001
**ASD Group**			
MT [s]	.131	12.324	.001
pV [mm/s]	.006	.489	.486
pA [mm/s^2]	.057	4.926	.029
pD [mm/s^2]	.010	.841	.362
$MaxD_X$ [m]	.009	.771	.383
$MaxD_Y$ [m]	.017	1.413	.238
$MaxD_Z$ [m]	.022	1.873	.175
TpV [s]	.003	.219	.641
T% Acc [%]	.244	26.498	<.001
T% Dec [%]	.242	26.235	<.001

Results report proportion of variance in the dependent variables that can be explained by the independent variable (R^2), F-statistic (F) and the p-value associated with it.

MT: movement time. pV: peak velocity. pA: peak acceleration. pD: peak deceleration. TpV: time to peak velocity. T%Acc: time % spent in acceleration. T% Dec: time % spent in deceleration. $MaxD_X$: Max Displacement along X axis. $MaxD_Y$: Max Displacement along Y axis. $MaxD_Z$: Max Displacement along Z axis.

Variables with R^2>=.29 and p-value<.05 are marked in ***bold***
