# Supplementary material for: Vitality form expression in autism
Source: Sci Rep. 2020 Oct 14;10:17182. doi: 10.1038/s41598-020-73364-x (PMC7560849; doi:10.1038/s41598-020-73364-x)
Supplement: Supplementary file 4 — Supplementary file4 [file 41598_2020_73364_MOESM4_ESM.pdf]

Supplementary Table S2. *How* condition taking into account the direction of the modulation.

Data for the two groups (ASD, TD) are reported as Median (IQR) values for each parameter. P-values of the inter-group analysis for the how_{Sign} index, reflecting the *how* condition taking into account the direction of modulation, are reported.

	how_{Sign} Median (IQR)		Inter-group
**parameters**	**TD**	**ASD**	(p-value TD vs. ASD)
MT [s]	0.276 (0.203)	0.103 (0.388)	**.002 ***
pV [mm/s]	- 1088.708 (867.488)	- 958.333 (791.992)	.336
pA [mm/s^2]	- 13870.608 (12199.448)	- 10713.875 (8774.4375)	.167
pD [mm/s^2]	- 23519.125 (19453.733)	- 15065.833 (12576.929)	**.031 ***
TpV [s]	0.006 (0.224)	-0.033 (0.310)	.458
T% Acc [%]	- 19.451 (30.534)	- 9.482 (26.0915)	.258
T% Dec [%]	15.170 (32.36)	9.467 (26.092)	.341
$MaxD_X$ [m]	-0.021 (0.048)	-0.040 (0.070)	**.039 ***
$MaxD_Y$ [m]	-0.036 (0.127)	-0.059 (0.131)	.601
$MaxD_Z$ [m]	-0.003 (0.036)	-0.002 (0.022)	.619

MT: movement time. pV: peak velocity. pA: peak acceleration. pD: peak deceleration. TpV: time to peak velocity. T%Acc: time spent in acceleration. T% Dec: time % spent in deceleration. $MaxD_X$: Max Displacement along X axis. $MaxD_Y$: Max Displacement along Y axis. $MaxD_Z$: Max Displacement along Z axis.

** . p-values TD vs. ASD Mann Whitney U test. <.05*

how_{Sign} = how_{Sign} index
